# Screening for Unruptured Intracranial Aneurysms in Autosomal Dominant Polycystic Kidney Disease: A Survey of 420 Nephrologists

**DOI:** 10.1371/journal.pone.0153176

**Published:** 2016-04-07

**Authors:** Adrien Flahault, Denis Trystram, Marie Fouchard, Bertrand Knebelmann, François Nataf, Dominique Joly

**Affiliations:** 1 Université Paris-Descartes; Faculté de Médecine; AP-HP; Service de Néphrologie, Hôpital Necker-Enfants Malades, Paris, France; 2 Université Paris-Descartes, INSERM UMR 894, Service de Neuroradiologie, Centre Hospitalier Sainte-Anne, Paris, France; 3 Université Paris-Descartes, INSERM UMR 894, Service de Neurochirurgie, Centre Hospitalier Sainte-Anne, Paris, France; Emory University, UNITED STATES

## Abstract

**Background:**

Despite a high prevalence of intracranial aneurysm (ICA) in autosomal dominant polycystic kidney disease (ADPKD), rupture events are rare. The current recommendations for ICA screening are based on expert opinions and studies with low levels of evidence.

**Objectives:**

The aim of our study was to describe the attitudes of practicing nephrologists in Europe towards screening for ICA using magnetic resonance angiography (MRA).

**Methods:**

We conducted a web-based survey among 1315 European French-speaking nephrologists and nephrology residents. An anonymous, electronic questionnaire including 24 independent questions related to ICA screening modalities, indications and participant profiles was sent by email between September and December 2014. Four hundred and twenty nephrologists (mostly from France) participated, including 31 nephrology residents; the response rate was 32%.

**Results:**

Systematic screening for ICA was advocated by 28% of the nephrologists. A family history of ICA rupture, sudden death, stroke and migraine were consensual indications for screening (> 90% of the panel). In other clinical situations largely not covered by the recommendations (pregnancy, nephrectomy, kidney transplantation, cardiac or hepatic surgery, uncontrolled hypertension, lack of familial ADPKD history, at-risk activity, tobacco use), the attitudes towards screening were highly divergent. ICA screening was influenced by nephrologists experience with ADPKD and by their practice setting. The majority of participants (57%) would not repeat a normal ICA screening. Only a few participants (22%) knew that non-contrast MRA was the reference diagnostic tool for ICA screening, whereas most participants thought that contrast enhancement was necessary to screen for ICA. The results from the nephrology residents were analyzed separately and yielded similar results.

**Conclusion:**

This practice survey revealed that most nephrologists follow the current recommendations for the initial screening of ICAs. However, more than a quarter of the panel was in favor of systematic ICA screening, most nephrologists did not know that contrast medium was not necessary to screen for ICA using MRA, and many areas of uncertainty remain.

## Introduction

Autosomal dominant polycystic kidney disease (ADPKD) is the most common hereditary kidney disease. The worldwide prevalence of ADPKD is estimated to be 1 per 400 to 1 per 1000 people [1,2]. ADPKD frequently leads to end-stage renal failure, accounting for approximately 10% of the renal replacement therapy (RRT) population in Europe [3] and 4.7% of the RRT population in the United States [4]. Several extra-renal manifestations are associated with ADPKD, including polycystic liver disease, mitral valve prolapse, and intracranial aneurysm (ICA) [5].

Intracranial aneurysms are acquired vascular lesions that have a prevalence of approximately 1.8% in the general population [6]. In the ADPKD population, the prevalence of unruptured ICA is between 9% and 12%, i.e., a prevalence ratio of 6.9 compared with the general population [7]. The ICA prevalence is even higher (approximately 22%) in cases of a positive family history of ICA. [8,9]

The main complication of ICA is rupture. The overall risk of rupture appears to be the same in ADPKD patients and in the general population. However, the mean age at ICA rupture is lower in the ADPKD population than in the general population (41 vs 51 years) [10].

Major risk factors for ICA rupture in the general population are aneurysm size and location, as well as smoking, high blood pressure and excessive alcohol intake [11]. In ADPKD patients, most ICA ruptures occur in association with large aneurysms, multiple and/or middle cerebral artery involvement, and poor blood pressure control [12]. Several studies have emphasized the familial clustering of ruptured ICAs in ADPKD [10,13,14]. Some authors have associated mutations in the 5’ region of PKD1 with an increased risk of ICA [15]; however, a large cohort study did not support this finding [16].

If diagnosed, unruptured ICA could be conservatively managed or could benefit from endovascular or surgical management depending on the aneurysm size, location, growth rate and to the patient’s age, comorbidities and family history [17].

There are no randomized, controlled studies assessing the benefit of screening for unruptured ICA in ADPKD patients. The majority of reviews on the subject, written by clinical experts in the field [5], as well as the KDIGO controversies conference [18], published in March 2015, suggest the following: (i) no systematic screening of ICA in ADPKD patients; (ii) targeted screening in patients with a good life expectancy who present with a family history of ICA or subarachnoid hemorrhage, patients with previous ICA rupture, those with high risk professions and anxious patients despite adequate information; (iii) the use of time-of-flight (TOF) magnetic resonance imagery (MRI) without gadolinium enhancement as the screening method of choice [19]; and (iv) rescreening at 5–10-year intervals in at-risk patients

Different opinions have also been published, advocating systematic screening for all patients with ADPKD [20] as well as screening before major elective surgery or renal transplantation. Apart from expert opinions, ICA screening practices in nephrology are mostly unknown. We set up an online survey to describe and analyze the current practices related to ICA screening among a large panel of European French-speaking nephrologists.

## Materials and Methods

### Participant selection

European French-speaking nephrologists who are members of the Société de Néphrologie (from France, Switzerland and Belgium) were contacted by email. Email addresses were obtained through the Société de Néphrologie’s directory and from several additional personal directories. In total, 1315 nephrologists were contacted by email between September and December 2014.

### Electronic form

The draft version of the form was tested in 27 nephrologists between July and September 2014 to check determine its practical application and make necessary improvements. There was no necessity to modify the form after this trial period.

The final version of the form (S1 and S2 Tables, English and French versions, respectively) was submitted to participants by email as a Google form document. Participation in the study was anonymous and voluntary, and the text accompanying the questionnaire (S1 File) indicated clearly that by submitting the questionnaire, he/she gave consent for data analysis and publication. The survey participants were informed of the fact that the data were to be used for research and that our objective was to publish the results. The participants were allowed to provide their email address. Those addresses and the questionnaire were automatically sent in separate folders to maintain the anonymity of the answers. Because the questionnaire was sent individually by email, we did not collect any supplemental data and did not seek approval from a data protection committee for this research. Completing the questionnaire, which consisted of 25 independent questions, required approximately 10 minutes. Because no investigation was made in patients, we did not seek approval from an ethics committee.

Sixteen questions addressed the relevance of ICA screening by MRI in different contexts. The participants were given the following five choices: “Unnecessary”, “Not very useful”, “Neutral”, “Appropriate”, “Necessary”. If the answer to the very first question of the survey, “Should a systematic baseline MRI be prescribed”, was “Necessary”, the participant was not asked specifically for other indications because the MRI would have been prescribed anyway.

In addition to possible indications concerning MRI screening, the participants were also asked if and when a normal MRI should be repeated, whether contrast medium should be used, and if extreme ages would influence their recommendations. Finally, data concerning the participant’s location, practice setting, year of medical degree and number of ADPKD patients in their active files were collected. For each of these variables, several classes were created to determine whether the answers to the questions varied depending on the medical experience of the nephrologist (medical degree before 1989; 1990 to 2004; 2005 to 2017), their practice setting (private practice/hospital setting), and their experience with polycystic kidney disease (0; 1 to 9; 10 to 49; 50 to 99; more than 100 PKD patients in their active files).

### Statistical analysis

The statistical analysis was conducted using R version 3.0.1 (R Development Core Team, 2005). Comparisons were made using Fisher’s exact test for comparing proportions and the Kruskal-Wallis rank sum test was used for multiple group comparisons. Differences were considered significant for a p-value < 0.05.

## Results

### Demographics

**Fig 1:**
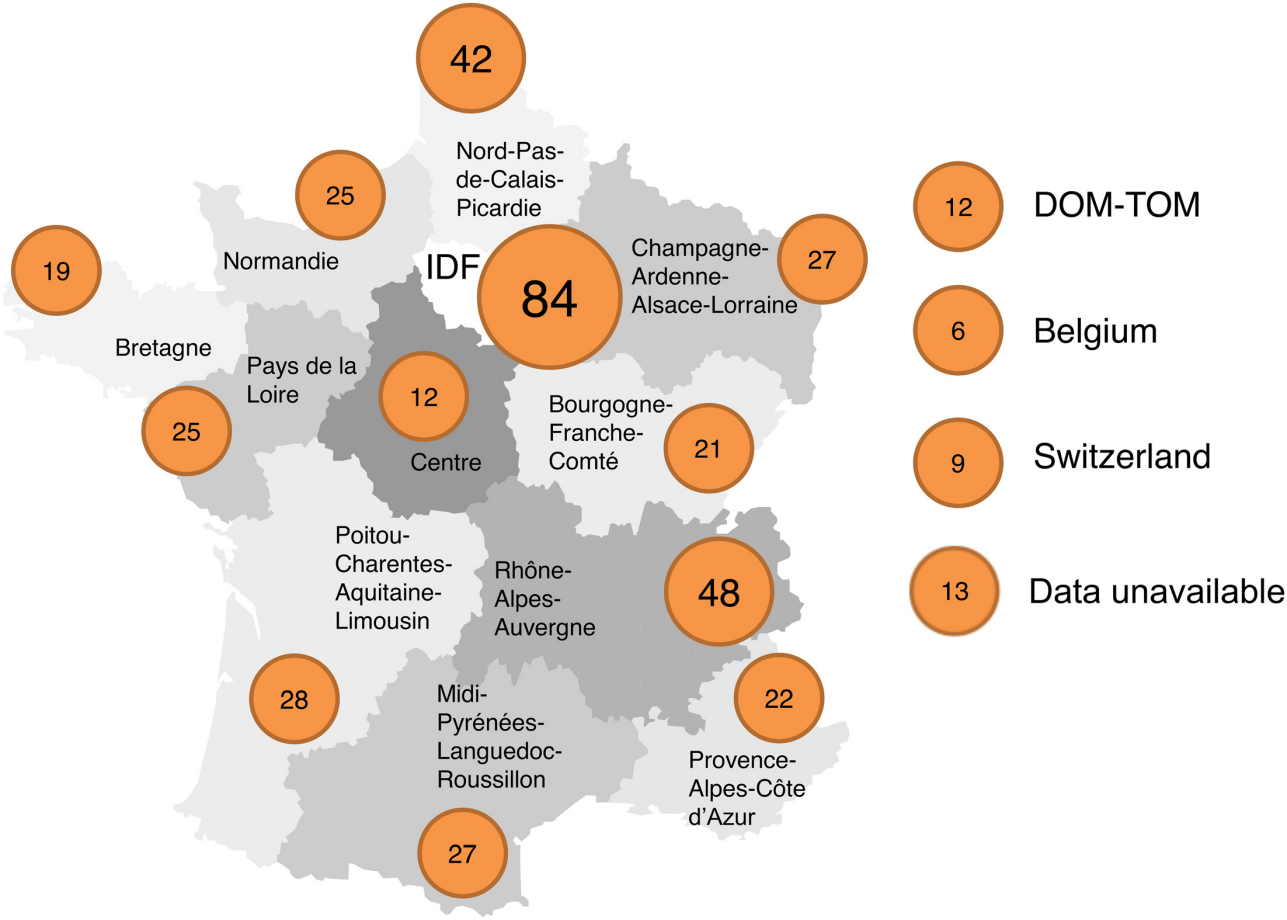
Distribution of nephrologists. Each circle contains the number of nephrologists (n = 420) participating in our study from each region. IDF: Ile-de-France. DOM-TOM: Départements et Territoires d’Outre-Mer. NA: not available. Map adapted from Vincent Viala with his authorization, http://vincentviala.com

**Fig 2:**
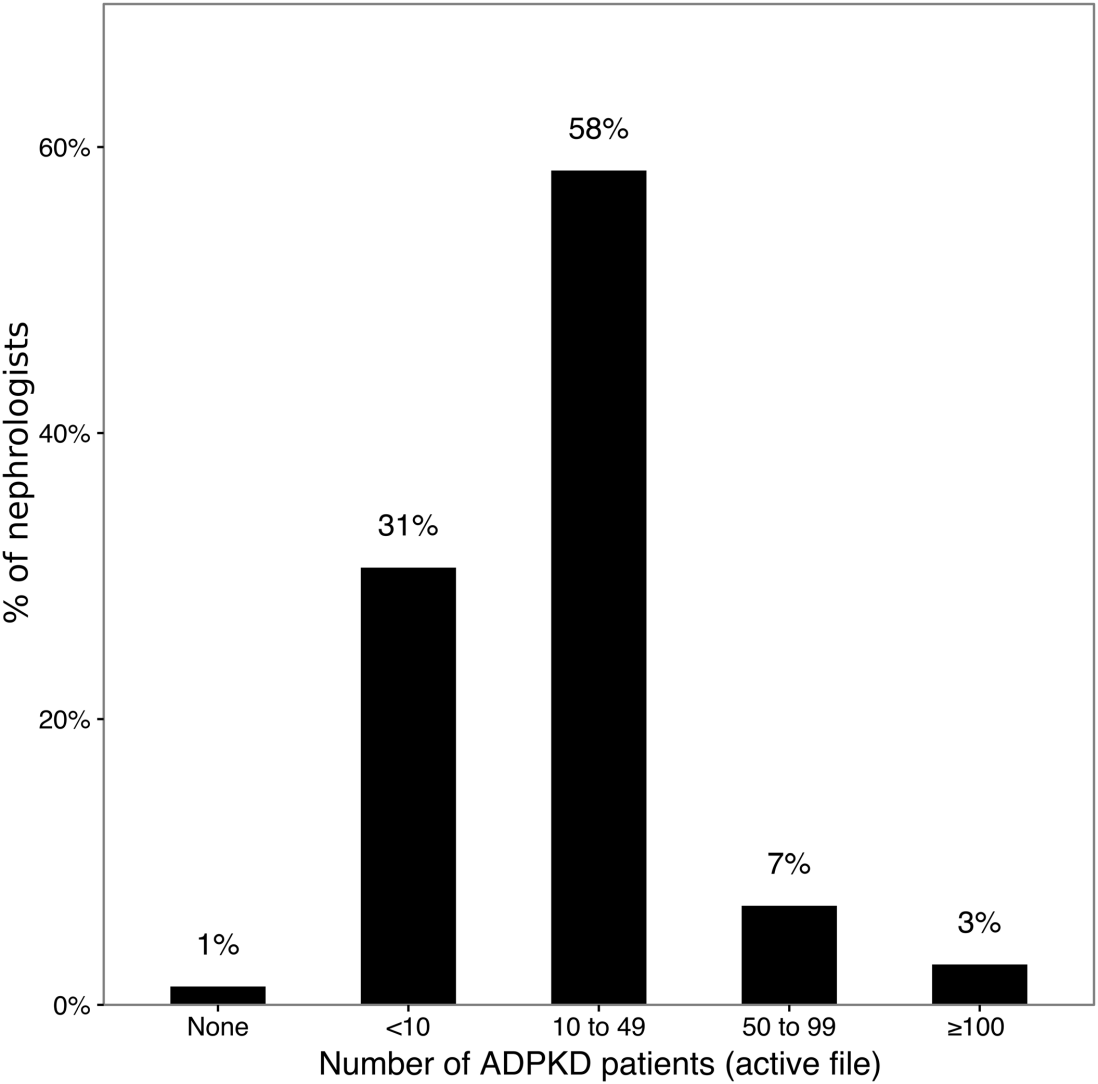
Current active file of ADPKD patients. N = 389; residents were excluded from this analysis because they did not personally follow any patients.

There were 420 participants in this study, including 31 nephrology residents. The results of the nephrology residents were analyzed separately. Fig 1 indicates the region of the geographic distribution of the participants, and S3 Table compares the distribution of nephrologists participating to our study with the number of registered nephrologists according to the French Ministry of Health. The geographic distribution of the participants was similar to the geographic distribution of nephrologists in France. Table 1 indicates the type of practice of the participants. In comparison with the global population of nephrologists in France, this study involved a higher proportion of physicians working in a public hospital and a lower proportion of physicians from private practices and residents. The year (or expected year) of medical degree ranged from 1962 to 2014. To determine their experience with polycystic kidney disease, the participants were asked to estimate their current active file of ADPKD patients. These results are depicted in Fig 2.

**Table 1:** Type of practice of the participants.

Nephrologists, by type of practice	Number of participants (%)	France (ministry of health)
*Non-teaching hospital*	*109 (26*,*0*)	
*Teaching hospital*	*184 (43*.*8)*	
**All public hospitals**	**293 (69.8)**	**908 (54.5%)**
*Private practice (nonprofit)*	*46 (11*.*0)*	
*Private practice (profit)*	*50 (11*.*9)*	
**All private practices**	**96 (22.9)**	**469 (28.1%)**
**Resident**	**31 (7.3)**	**290 (17.4%)**
**Total**	**420**	**1667**

Including the nephrologists in France in 2014, according to the French Ministry of Health.

### Routine baseline screening

**Fig 3:**
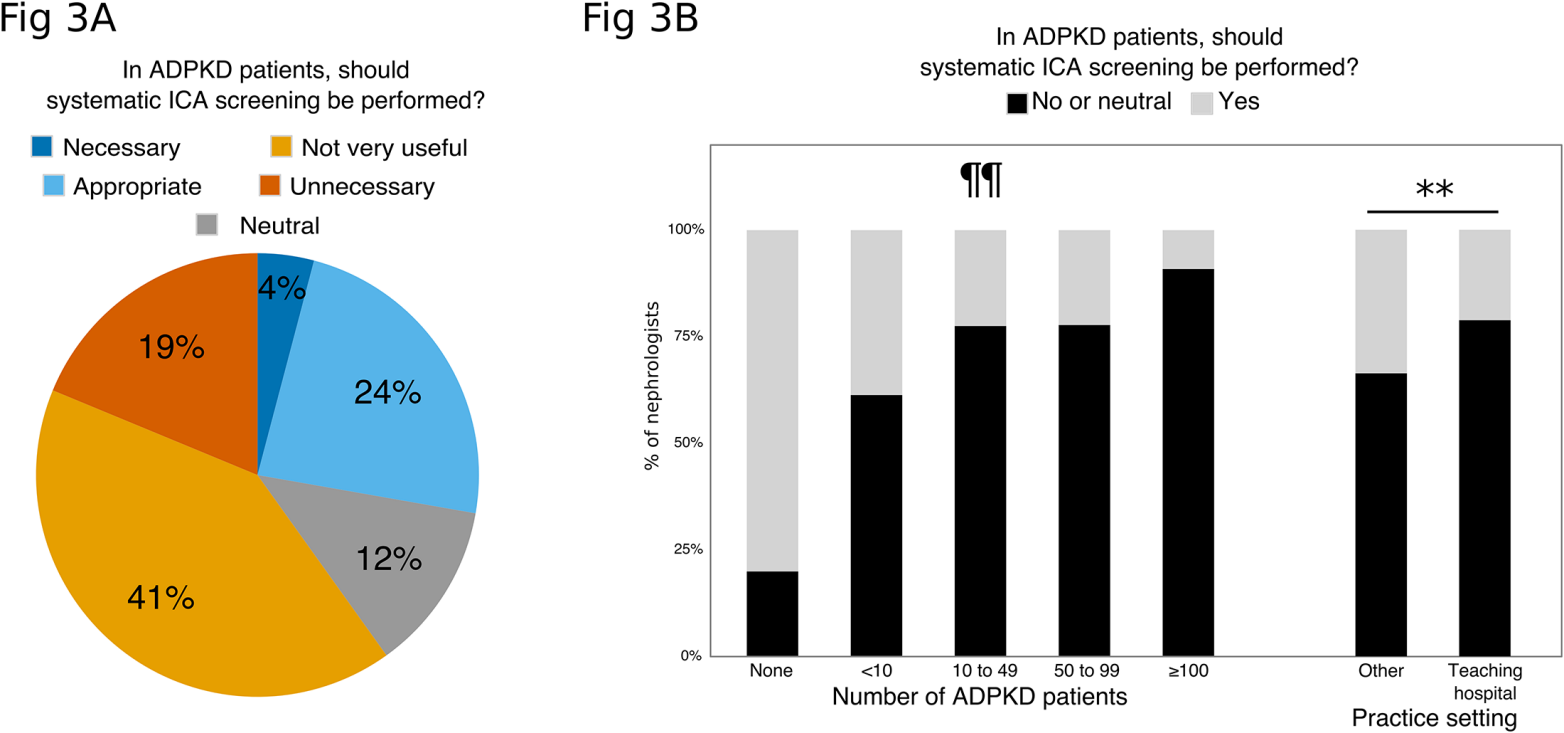
**a. Attitudes towards systematic ICA screening.** N = 389; residents were excluded from this analysis. **b**. **Systematic screening (black bars) according to the number of ADPKD patients actually in follow-up and work setting.** N = 389; residents were excluded from this analysis. Comparison of the proportion of respondents in favor of systematic screening according to the number of ADPKD patients in follow up: ** p<0.01 (Kruskal-Wallis test). Comparison of the proportion of respondents in favor of systematic screening according to their work setting: ¶¶ p<0.001 (Fisher exact test).

The first question to the participants addressed their attitude towards a systematic baseline screening of ICA using MRI for all ADPKD patients. As shown in Fig 3a, 28% of the panel found this attitude either “Necessary” (n = 16; 4%) or “Appropriate” (n = 92; 24%), and 60% found this attitude either “Not very useful” or “Unnecessary”.

### Indications for targeted screening

**Fig 4:**
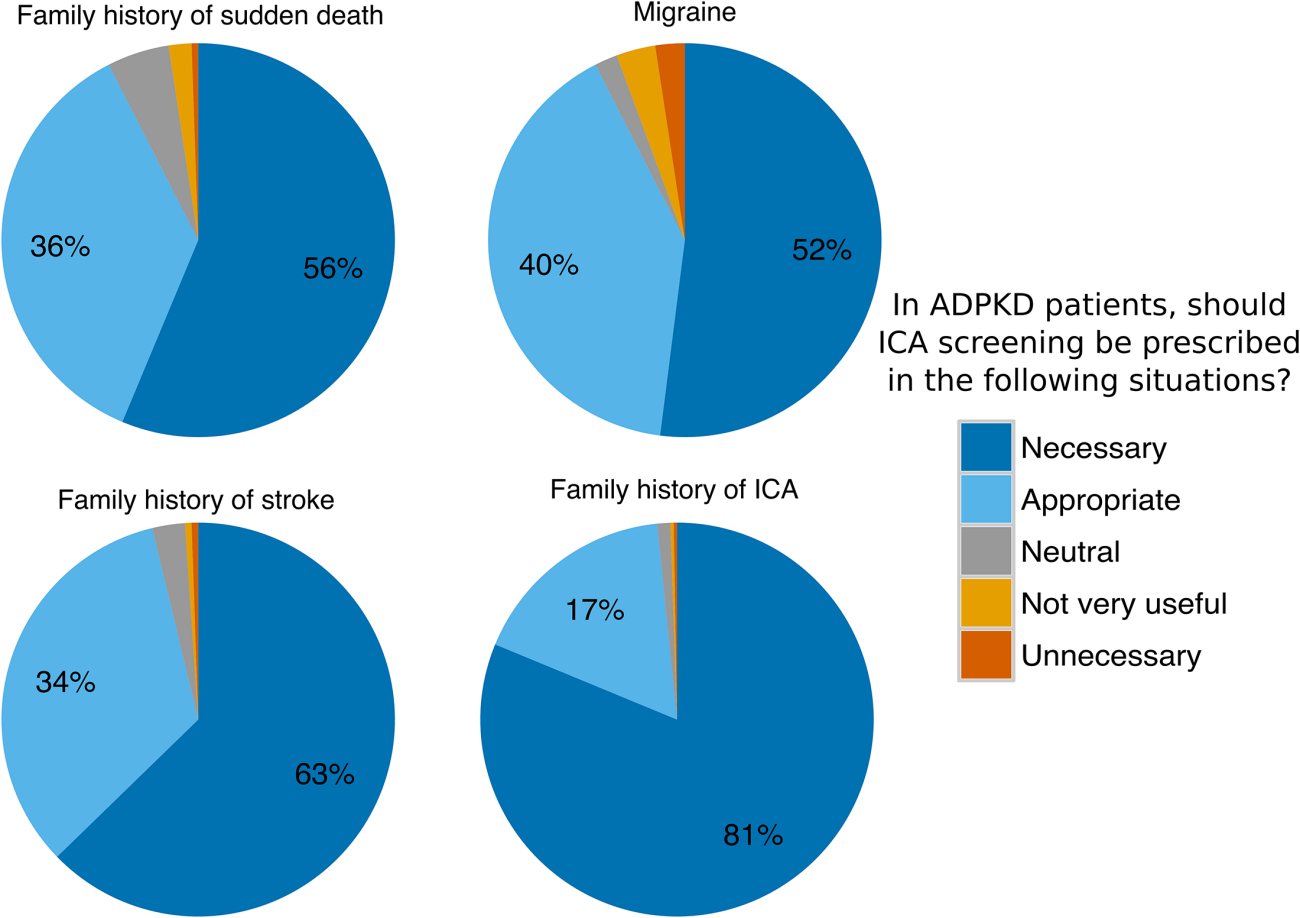
Attitude towards ICA screening: consensual indications. N = 373; residents were excluded from this analysis.

When systematic screening was not considered “necessary”, the nephrologist was questioned regarding the potential interest of ICA screening in several specific situations. There was a large consensus to recommend screening in cases of a family history of ICA rupture (98%), a family history of sudden death (92%) or a family history of stroke of an unknown etiology before 65 years of age (96%), and in cases of recurrent headaches resembling migraine (92%) (Fig 4).

**Fig 5:**
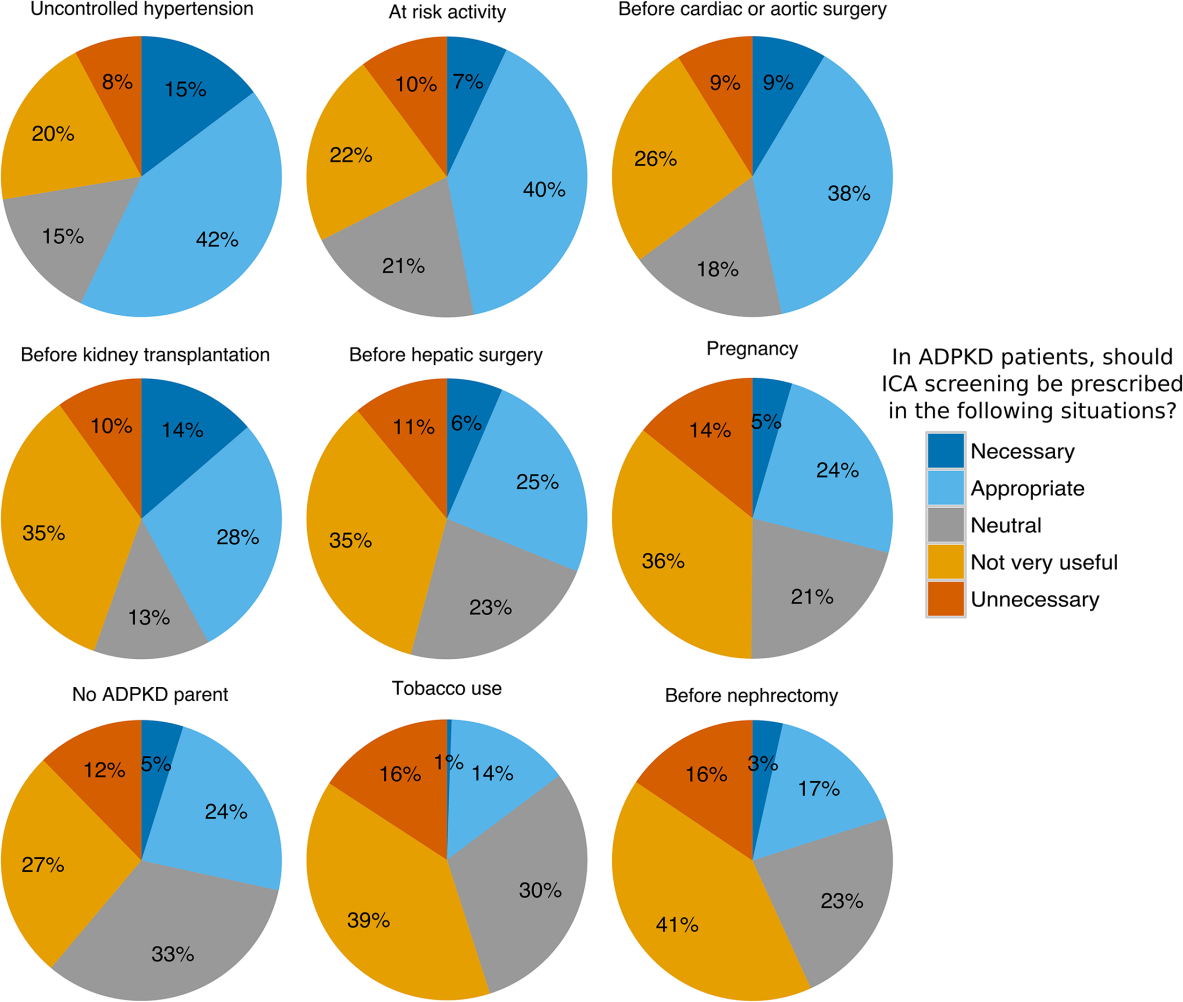
Attitude towards ICA screening: non-consensual indications. N = 373; residents were excluded from this analysis.

As shown Fig 5, the opinions regarding ICA screening were much more divided for nine other frequent situations, including pregnancy, cardiovascular surgery, hepatic surgery, kidney transplantation (Yes: 42%, No: 45%, Neutral: 13%), and lack of PKD family history (Yes: 40%, No: 27%, Neutral: 33%).

### Contrast enhancement and control imaging

**Fig 6:**
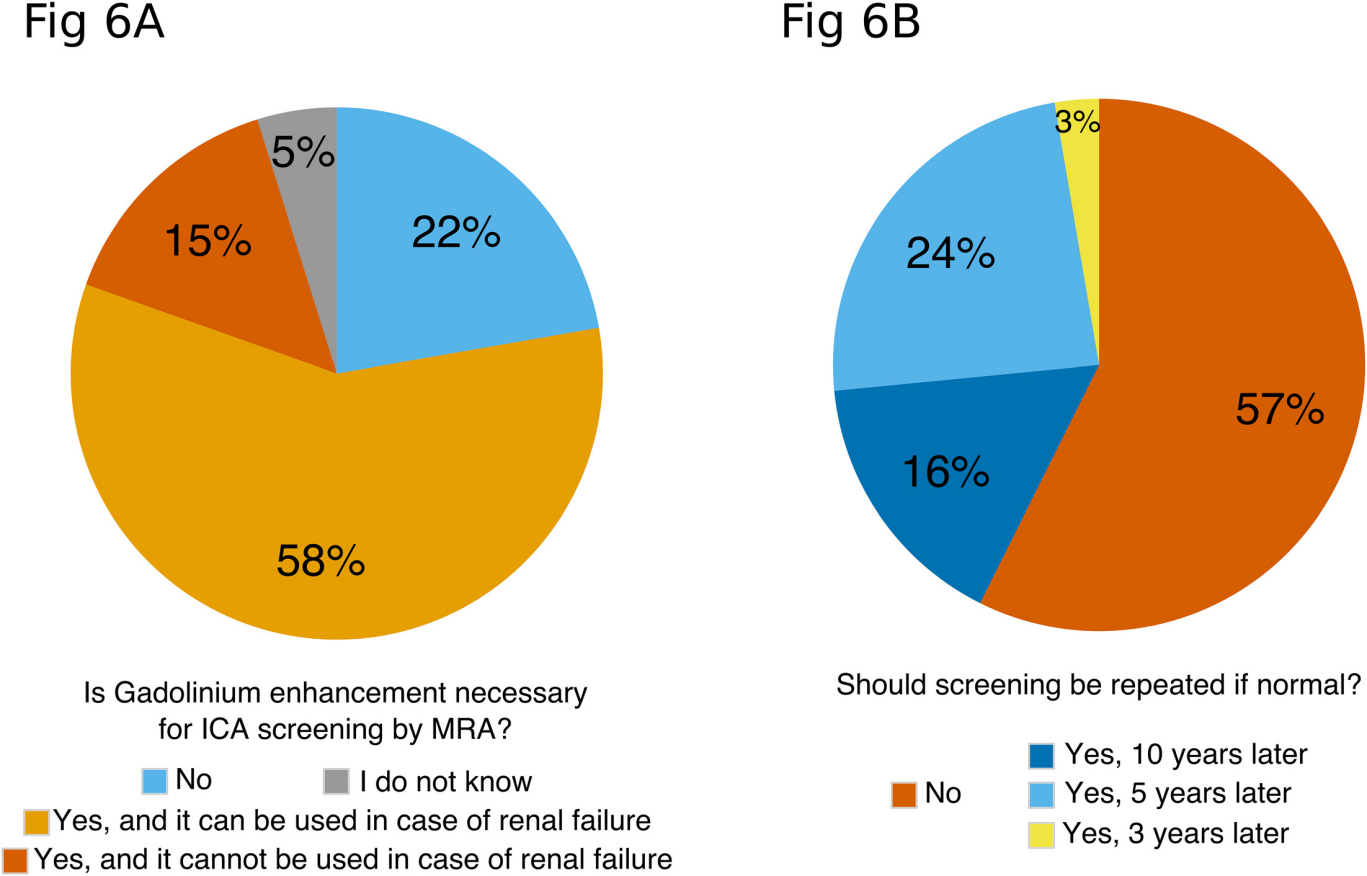
**a. Is gadolinium enhancement necessary for ICA screening by MRA?** N = 373; residents were excluded from this analysis. **b. Should screening be repeated if normal?** N = 373; residents were excluded from this analysis.

Participants were asked if gadolinium enhancement using gadoteric acid was necessary to screen for ICA by MRI (Fig 6a). Few participants (n = 83; 22%) knew that gadolinium enhancement was not necessary, and 28 (5%) did not express an opinion. Most participants (n = 272; 73%) thought contrast enhancement was essential, and some (n = 55; 15%) also thought that severe renal failure would both prevent using gadolinium and performing ICA screening.

For patients with an indication for ICA screening, the participants were also asked if screening should be repeated if normal (Fig 6b). The KDIGO panel recommends repeating ICA screening every 5 to 10 years. Among participants in this study, most (n = 214; 57%) considered there was no indication to repeat screening, 60 (16%) would prescribe a control MRI 10 years later, 89 (24%) would prescribe a control MRI 5 years later and 10 (3%) would prescribe a control MRI 3 years later.

### Patient information

If ICA screening was not indicated, the nephrologist was asked what information concerning the risk of ICA rupture was given to the patient. Few participants (n = 45; 12%) would not give any information to the patient because they were not willing to worry the patient. Most participants (n = 197; 53%) would address the matter on a case-by-case basis, according to the patient’s demands. Many participants (n = 131; 35%) would systematically inform the patient of the risk of ICA rupture and of the actions to take in case of suggestive symptoms.

### Patient requests for ICA screening

Another difficult situation is when the patient requests ICA screening although the clinician did not agree with the indication to screen. The KDIGO panel recommends, in this situation, to prescribe the MRI after appropriate information is given. This behavior was also adopted by 243 (65%) nephrologists in our study, whereas 24 (6%) would systematically refuse to prescribe an MRI, and 106 (28%) would systematically accept to prescribe without any attempt to convince the patient of the uselessness of screening.

### Factors influencing screening policy

The more experience that the nephrologists had with PKD (as determined by the number of ADPKD patients in their active file), the less they were likely to prescribe systematic MRI screening (18% favoring systematic baseline screening in nephrologists with an active file of more than 50 ADPKD patients versus 33%, p<0.001, Fig 3b), pre-kidney transplantation screening (28% versus 44%, p = 0.01) or screening before or during pregnancy (5% versus 31%, p = 0.006).

Physicians working in university-based settings were less likely to prescribe systematic MRI baseline screening (21% favoring systematic baseline screening in university-based setting versus 34%, p = 0.005, Fig 3b) and more likely to repeat a normal baseline screening (51% versus 35%, p = 0.002). There was no influence of practice setting on any other item of the questionnaire.

### Collegiality of screening practice

Participants were asked if their ICA screening policy for ADPKD patients was the same as the other nephrologists in their hospital or practice. The majority of the participants (n = 201, 52%) did not know, 150 (39%) had a similar screening policy and 38 (10%) had a different policy.

### Residents

Responses concerning the residents were analyzed separately in this study. Ten (32%) of the residents were in favor of routine baseline screening in all ADPKD patients; 66% of the residents would not repeat screening if the baseline screening is normal, and only 17% of the residents knew that contrast enhancement was not necessary to perform screening for ICA using MRA.

## Discussion

This survey is the first to explore the attitude concerning ICA screening in ADPKD patients among a large panel of nephrologists.

The results of this investigation deserve to be confronted with the KDIGO controversies conference text, which includes several recommendations for ICA screening in ADPKD. KDIGO recommendations were published shortly after our survey was conducted [18]. These recommendations might ultimately increase the consistency of ICA screening practices among nephrologists throughout the world. These recommendations, however, are not based on new evidence and are very similar to positions previously taken by several leading experts in the field.

The KDIGO panel does not recommend systematic screening for ICA. The majority of the participants in our study agreed with this attitude; however, 28% of the participants considered that systematic MRI baseline screening for ICA should be performed in all ADPKD patients. There is currently little clinical evidence to advocate for or against this attitude. No large-scale, controlled study has studied the benefits and risks of targeted screening versus systematic screening in ADPKD. The KDIGO panel indicates that systematic screening yields mostly small ICAs with a low risk of rupture, for which prophylactic repair might be risky. Some authors advocate for widespread ICA screening in ADPKD patients [20], arguing that (i) the risk of presenting with ICA is elevated in ADPKD patients and (ii) guidelines were recently established to manage unruptured ICA [17], with a good level of proof. These guidelines provide recommendations for conservative management, endovascular or neurosurgical treatment according to the risk of rupture, although the guidelines were not established specifically for the ADPKD population.

There is evidence of a much higher risk of ICA rupture in patients with a family history of ICA rupture [8,9]. We observed a widespread consensus among the respondents in our study (98%), in agreement with the recommendations published in the literature and by the KDIGO panel. There was also a consensus regarding clinical situations such as a family history of stroke (96%) or sudden death (92%). The participants in our study were also largely in favor of screening for ICA in cases of recurrent, typical migraine headaches (92%). Unruptured ICAs are typically asymptomatic, and there is no evidence that ADPKD patients with migraine are more at risk of ICA. This specific indication for screening is not discussed by the KDIGO.

The other known risk factors for ICA rupture, although not proven in the ADPKD population, are smoking and high blood pressure. The KDIGO panel does not recommend systematic screening in this situation, although it strongly recommends smoking cessation and control of cardiovascular risk factors. A majority of the respondents in our study did not recommend systematic ICA screening in cases of smoking, although more than half of the nephrologists would screen for ICA in cases of uncontrolled and severe hypertension.

Another subject of diverging opinion among the nephrologists in this survey was the necessity to perform ICA screening prior to kidney transplantation. The literature is divergent on this point. In a recent review [21], Kanaan et al. advocate against systematic pre-transplant screening because of the following reasons: (i) the mean age at ICA rupture in ADPKD patients is approximately 41 years, which is younger than the age at transplantation for this population; therefore, in at risk patients, ICA rupture would probably occur before transplantation; (ii) findings from survival analyses and cohort studies indicate that the risk of stroke only marginally affects the morbidity and mortality of patients with ADPKD after kidney transplantation [22]; and (iii) there is a significantly lower risk of hemorrhagic stroke in patients who have undergone renal transplantation compared with those who remain on hemodialysis [23].

There is not much evidence, regarding the risk of ICA rupture in other clinical situations that could happen at a younger age, such as pregnancy and child delivery, major elective aortic or liver surgery.

This survey shows that few participants are aware that time-of-flight MRI without contrast medium is the gold standard for ICA screening [19]. Some participants would also both contraindicate gadolinium and MRI screening in cases of severe renal failure. This finding underlines the necessity to better inform the nephrology community on the fact that contrast medium is not necessary to screen for ICA. Additionally, more than half of participants would not repeat screening, although it has been shown that ICAs might appear on repeat imaging during adulthood [24]. A subset analysis, conducted among the residents who participated to our study, showed that residents were equally misinformed concerning the screening method and the need to repeat screening.

Several physician-dependent covariates were evaluated in regards to the attitudes towards ICA screening. Our data mainly indicate that the most experienced physicians in ADPKD, as well as physicians working in teaching hospitals, were less likely to prescribe systematic MRI screening. This finding could reflect a better knowledge of the recommendations and a different perception of the cost and the expected benefits of screening given the incidence of ICA complications in the ADPKD population.

Our study has several limitations. Although most of the licensed nephrologists were contacted, we obtained responses from only approximately one-fourth of the nephrologists in our country. We managed to obtain a geographically representative panel, although physicians from hospital-based settings were slightly over-represented. Our results do not reflect the medical practice of nephrologists from other countries, in which attitudes towards ICA screening might differ. More generally, the attitudes depicted in this survey result from a virtual reflection; it is therefore possible that attitudes towards ICA screening in ADPKD might differ in clinical situations from real life. However, the use of this methodology has been validated for measuring the quality of medical practice and adherence to guidelines [25,26]. We did not ask participants about the proportion of ADPKD patients to total patients, and we did not differentiate pre-ESRD patients from ESRD patients and transplant recipients. Including this information would have perhaps allowed for a better identification of nephrologists specialized in ADPKD.

Given the lack of solid evidence-based guidelines and the sometimes conflicting opinions published in the literature, this survey and its methodology underscore specific difficulties and open research perspectives.

Our results emphasize the following: (i) the need to better inform nephrologists about ICA screening, including the fact that the use of gadolinium-based contrast medium is not necessary to screen for ICA using MRA, and the need for repeat imaging even after a normal initial screening; (ii) the large adhesion of nephrologists to consensual indications for ICA screening; (iii) the widely divergent attitudes in clinical situations to which the physician is frequently exposed, which are not addressed in the current recommendations; and (iv) the need for a large-scale study comparing the efficacy and safety of a targeted screening compared with a systematic ICA screening in the ADPKD population to establish future recommendations with a sufficient level of evidence.

## Supporting Information

S1 File Letter to the Participant. Letter sent by email to each participant explaining the objective of the study; the original French version and a translation in English are displayed.
(DOCX)

S1 Table Electronic form (English version). Items of the questionnaire sent to the participant, translated in English.
(DOCX)

S2 Table Electronic form (French version). Items of the questionnaire sent to the participant, in the original (French) version.
(DOCX)

S3 Table Geographic distribution of the participants and of the nephrologists in France in 2014 according to the French Ministry of Health. Residents were excluded from this table. NA: not applicable. P-value = 0.3.
(DOCX)
