# Supplementary material for: Screening for Unruptured Intracranial Aneurysms in Autosomal Dominant Polycystic Kidney Disease: A Survey of 420 Nephrologists
Source: PLoS One. 2016 Apr 7;11(4):e0153176. doi: 10.1371/journal.pone.0153176 (PMC4824518; doi:10.1371/journal.pone.0153176)
Supplement: S1 File — Letter sent by email to each participant explaining the objective of the study; the original French version and a translation in English are displayed. (DOCX) [file pone.0153176.s001.docx]

**S1 File. Letter to the Participant**

*(French)*

Cher Monsieur/Madame,

**chez qui est il utile de dépister les anévrismes cérébraux dans la polykystose dominante ?**

Cette question est difficile et non consensuelle, si bien qu’il nous a semblé utile d’effectuer une "enquête sur les pratiques" auprès des membres de la communauté néphrologique francophone.

Les résultats de cette enquête seront utiles à ma thèse d'exercice réalisée sous la direction du Pr Joly en vue d'une publication scientifique, ainsi qu’à un consensus d'experts et à un éventuel travail prospectif.

C’est facile : cases à cocher (10 min ± commentaires éventuels)

- cliquer sur le lien ci dessous

- globalement, dites ce que vous faites personnellement, dans la vie réelle

- les réponses sont anonymisées

- la dernière case permet toutefois de laisser votre email pour être au courant de la suite

<https://docs.google.com/forms/d/1PMJ8BiuuYJTXTQwvoUgXn8o5OSoRZ0mmBppv2kajhnA/viewform?usp=send_form>

Merci vivement par avance de votre aide.

Cordialement,

Adrien Flahault

DES néphrologie

*(English)*

Dear Sir/Madam,

in whom is it necessary to screen for intracranial aneurism in autosomal dominant polycystic kidney disease ?

This is a difficult and non consensual question, we therefore found useful to study the practices regarding this question among members of the french-speaking community of nephrologists.

The results of this study will be useful to the obtention of my medical doctorate under the direction of Prof. D. Joly and will hopefully lead to scientific publication. We hope the results will help to establish an expert consensus and lead to a prospective study.

It's easy. Checkboxes (10 min, possible comments)

- click the link below

- indicate what you usually do, personally, in the real life

- the answers are anonymous

- you can leave you email in order to be informed of the results

<https://docs.google.com/forms/d/1PMJ8BiuuYJTXTQwvoUgXn8o5OSoRZ0mmBppv2kajhnA/viewform?usp=send_form>

Thank you in advance for your help.

Sincerely,

Adrien Flahault

Resident in Nephrology
