# Supplementary material for: Screening for Unruptured Intracranial Aneurysms in Autosomal Dominant Polycystic Kidney Disease: A Survey of 420 Nephrologists
Source: PLoS One. 2016 Apr 7;11(4):e0153176. doi: 10.1371/journal.pone.0153176 (PMC4824518; doi:10.1371/journal.pone.0153176)
Supplement: S1 Table — Items of the questionnaire sent to the participant, translated in English. (DOCX) [file pone.0153176.s002.docx]

**S1 Table**. Electronic form (English version)

| 1. Should a systematic baseline MRI be prescribed? | Unnecessary ☐ Not very useful ☐ Neutral ☐ Appropriate ☐ Necessary ☐ |
| --- | --- |
| 1. Screening before cardiac or aortic surgery | Unnecessary ☐ Not very useful ☐ Neutral ☐ Appropriate ☐ Necessary ☐ |
| 1. Screening before hepatic surgery | Unnecessary ☐ Not very useful ☐ Neutral ☐ Appropriate ☐ Necessary ☐ |
| 1. Screening before nephrectomy | Unnecessary ☐ Not very useful ☐ Neutral ☐ Appropriate ☐ Necessary ☐ |
| 1. Screening before kidney transplantation | Unnecessary ☐ Not very useful ☐ Neutral ☐ Appropriate ☐ Necessary ☐ |
| 1. Screening before or in case of pregnancy | Unnecessary ☐ Not very useful ☐ Neutral ☐ Appropriate ☐ Necessary ☐ |
| 1. Screening in case of at an risk occupation or activity | Unnecessary ☐ Not very useful ☐ Neutral ☐ Appropriate ☐ Necessary ☐ |
| 1. Screening in case of smoking | Unnecessary ☐ Not very useful ☐ Neutral ☐ Appropriate ☐ Necessary ☐ |
| 1. Screening in case of uncontrolled hypertension | Unnecessary ☐ Not very useful ☐ Neutral ☐ Appropriate ☐ Necessary ☐ |
| 1. Screening in case of recurrent migraine | Unnecessary ☐ Not very useful ☐ Neutral ☐ Appropriate ☐ Necessary ☐ |
| 1. Screening in case of family history of ICA rupture | Unnecessary ☐ Not very useful ☐ Neutral ☐ Appropriate ☐ Necessary ☐ |
| 1. Screening in case of family history of sudden death | Unnecessary ☐ Not very useful ☐ Neutral ☐ Appropriate ☐ Necessary ☐ |
| 1. Screening in case of family history of stroke before 65 years of age | Unnecessary ☐ Not very useful ☐ Neutral ☐ Appropriate ☐ Necessary ☐ |
| 1. Screening in case of family history of stroke after 65 years of age | Unnecessary ☐ Not very useful ☐ Neutral ☐ Appropriate ☐ Necessary ☐ |
| 1. Screening in the absence of family history of ADPKD | Unnecessary ☐ Not very useful ☐ Neutral ☐ Appropriate ☐ Necessary ☐ |
| 1. Screening in case family history is unknown | Unnecessary ☐ Not very useful ☐ Neutral ☐ Appropriate ☐ Necessary ☐ |
| 1. If normal, should ICA screening be repeated? | - No |
|  | - Yes, 10 years later |
|  | - Yes, 5 years later |
|  | - Yes, 3 years later |
| 1. Is gadolinium enhancement using gadoteric acid necessary to screen for ICA by MRI? | - Yes, and it contra-indicates its use in case of severe renal failure |
|  | - Yes, but its use is permitted even in case of severe renal failure |
|  | - No, it is optional |
|  | - I do not know |
| 1. If ICA screening was not indicated, what do you do if your patient requests screening anyway? | - I systematically prescribe the MRI |
|  | - I prescribe the MRI after appropriate information |
|  | - I refuse to prescribe the MRI |
| 1. If ICA screening was not indicated, information concerning the risk of ICA rupture do you give your patient? | - None, not to worry him |
|  | - I systematically inform the patient of the risk of ICA rupture and of the actions to take in case of suggestive symptoms |
|  | - I address the matter on a case-by-case basis, according to the patient’s demands |
| 1. How would you describe your ICA screening policy for ADPKD patients compared to the other nephrologists in your hospital or practice? | - Similar |
|  | - Different |
|  | - I do not know |
| 1. How many ADPKD patients do you currently follow? | - None |
|  | - < 10 |
|  | - 10 to 49 |
|  | - 50 to 99 |
|  | - > 100 |
| 1. What is your practice setting? | - University public hospital |
|  | - Public hospital |
|  | - Private practice (associative) |
|  | - Private practice (liberal) |
|  | - Resident in nephrology |
| 1. Year of medical degree |  |
| 1. Location of practice |  |
