# Supplementary material for: Screening for Unruptured Intracranial Aneurysms in Autosomal Dominant Polycystic Kidney Disease: A Survey of 420 Nephrologists
Source: PLoS One. 2016 Apr 7;11(4):e0153176. doi: 10.1371/journal.pone.0153176 (PMC4824518; doi:10.1371/journal.pone.0153176)
Supplement: S2 Table — Items of the questionnaire sent to the participant, in the original (French) version. (DOCX) [file pone.0153176.s003.docx]

**S2 table**. Electronic form (French version)

| 1. Faut-il proposer une angio-IRM cérébrale (ARM) de référence à tous les patients polykystiques? | Pas du tout approprié ☐ Peu utile ☐ Neutre ☐ Approprié ☐ Indispensable ☐ |
| --- | --- |
| Faut il: |  |
| 1. Proposer une angio-IRM cérébrale avant une chirurgie cardiaque ou aortique | Pas du tout approprié ☐ Peu utile ☐ Neutre ☐ Approprié ☐ Indispensable ☐ |
| 1. Proposer une angio-IRM cérébrale avant une chirurgie hépatique (hépatectomie partielle, greffe) | Pas du tout approprié ☐ Peu utile ☐ Neutre ☐ Approprié ☐ Indispensable ☐ |
| 1. Proposer une angio-IRM cérébrale avant une néphrectomie | Pas du tout approprié ☐ Peu utile ☐ Neutre ☐ Approprié ☐ Indispensable ☐ |
| 1. Proposer une angio-IRM cérébrale dans le cadre d'un bilan pré-greffe rénale | Pas du tout approprié ☐ Peu utile ☐ Neutre ☐ Approprié ☐ Indispensable ☐ |
| 1. Proposer une angio-IRM cérébrale en prévision, ou à la découverte, d'une grossesse | Pas du tout approprié ☐ Peu utile ☐ Neutre ☐ Approprié ☐ Indispensable ☐ |
| 1. Proposer une angio-IRM cérébrale dans le cadre d'une profession ou d’une activité à risque | Pas du tout approprié ☐ Peu utile ☐ Neutre ☐ Approprié ☐ Indispensable ☐ |
| 1. Proposer une angio-IRM cérébrale en cas de tabagisme actif | Pas du tout approprié ☐ Peu utile ☐ Neutre ☐ Approprié ☐ Indispensable ☐ |
| 1. Proposer une angio-IRM cérébrale en cas d'hypertension artérielle sévère mal contrôlée | Pas du tout approprié ☐ Peu utile ☐ Neutre ☐ Approprié ☐ Indispensable ☐ |
| 1. Proposer une angio-IRM cérébrale en cas de céphalées récurrentes d’allure migraineuse | Pas du tout approprié ☐ Peu utile ☐ Neutre ☐ Approprié ☐ Indispensable ☐ |
| 1. Proposer une angio-IRM cérébrale en cas d'antécédent familial de rupture d'anévrysme | Pas du tout approprié ☐ Peu utile ☐ Neutre ☐ Approprié ☐ Indispensable ☐ |
| 1. Proposer une angio-IRM cérébrale en cas d'antécédent familial de mort subite | Pas du tout approprié ☐ Peu utile ☐ Neutre ☐ Approprié ☐ Indispensable ☐ |
| 1. Proposer une angio-IRM cérébrale en cas d'antécédent familial d'accident vasculaire cérébral à un âge jeune (< 65 ans) | Pas du tout approprié ☐ Peu utile ☐ Neutre ☐ Approprié ☐ Indispensable ☐ |
| 1. Proposer une angio-IRM cérébrale en cas d'antécédent familial d'accident vasculaire cérébral après 65 ans | Pas du tout approprié ☐ Peu utile ☐ Neutre ☐ Approprié ☐ Indispensable ☐ |
| 1. Proposer une angio-IRM cérébrale « de principe» chez un patient dont les parents ne sont pas atteints de polykystose (par exemple, mutation de novo) | Pas du tout approprié ☐ Peu utile ☐ Neutre ☐ Approprié ☐ Indispensable ☐ |
| 1. Proposer une angio-IRM cérébrale « de principe» chez un patient dont les antécédents familiaux ne sont pas connus ? | Pas du tout approprié ☐ Peu utile ☐ Neutre ☐ Approprié ☐ Indispensable ☐ |
| 1. Si l'IRM proposée ne décèle aucun anévrisme, proposez vous une angio-IRM cérébrale de contrôle? | - Non |
|  | - Oui, 10 ans plus tard |
|  | - Oui, 5 ans plus tard |
|  | - Oui, 3 ans plus tard |
| 1. Pour vous, est-ce que l'injection de produit de contraste (gadolinium, Dotarem®)? | - Est indispensable, mais est contre-indiquée chez l'insuffisant rénal sévère |
|  | - Est indispensable et n’est pas contre-indiquée chez l'insuffisant rénal sévère |
|  | - Est facultative |
|  | - Je ne sais pas |
| 1. Supposons que vous n’ayez pas retenu l’indication d’un dépistage d’anévrisme chez un patient; que faites vous si le patient demande une angio-IRM cérébrale de dépistage? | - Je la prescris systématiquement |
|  | - J'essaie de le convaincre de son inutilité, mais la prescris si la demande est insistante |
|  | - Je refuse |
| 1. Supposons que vous n’ayez pas retenu l’indication d’un dépistage d’anevrisme chez un patient; quelles informations lui donnez vous sur le risque de rupture d’anévrisme? | - Aucune, car il est inutile de l’inquiéter |
|  | - J’évoque tout de même systématiquement cette complication et la conduite à tenir en cas de signes évocateurs de rupture |
|  | - J'évoque cette complication au cas par cas, en fonction de la demande du patient |
| 1. Pensez vous que les collègues avec qui vous travaillez ont une politique de prescription des angio IRM dans la polykystose: | - Similaire |
|  | - Différente |
|  | - Je ne sais pas |
| 1. Combien de patients différents suivez-vous en consultation pour une polykystose rénale (file active)? | - Aucun |
|  | - < 10 |
|  | - 10 à 49 |
|  | - 50 à 99 |
|  | - > 100 |
| 1. Mode d’exercice principal | - Centre Hospitalo- Universitaire |
|  | - Centre Hospitalier général |
|  | - Privé associatif |
|  | - Privé libéral |
|  | - Interne |
| 1. Année de thèse d'exercice (ou année prévue de soutenance de thèse): |  |
| 1. Département d'exercice (ou pays): |  |
