# Supplementary material for: Screening for Unruptured Intracranial Aneurysms in Autosomal Dominant Polycystic Kidney Disease: A Survey of 420 Nephrologists
Source: PLoS One. 2016 Apr 7;11(4):e0153176. doi: 10.1371/journal.pone.0153176 (PMC4824518; doi:10.1371/journal.pone.0153176)
Supplement: S3 Table — Residents were excluded from this table. NA: not applicable. P-value = 0.3. (DOCX) [file pone.0153176.s004.docx]

**S3 Table**. **Geographic distribution of the participants and of the nephrologists in France in 2014 according to the French Ministry of Health.** Residents were excluded from this table. NA: not applicable. P-value = 0.3

| Nephrologists, by region or country | Number of participants in this study (%) | Number of nephrologists (Ministry of Health) (%) |
| --- | --- | --- |
| Alsace | 10 (3) | 51 (3) |
| Aquitaine | 15 (4) | 64 (4) |
| Auvergne | 7 (2) | 28 (2) |
| Basse-Normandie | 10 (3) | 28 (2) |
| Bourgogne | 12 (3) | 33 (2) |
| Bretagne | 18 (5) | 68 (4) |
| Centre | 12 (3) | 57 (4) |
| Champagne-Ardenne | 6 (2) | 26 (2) |
| Corse | 0 (0) | 5 (0) |
| Franche-Comté | 8 (2) | 23 (2) |
| Haute-Normandie | 15 (4) | 46 (3) |
| Ile-de-France | 67 (19) | 335 (22) |
| Languedoc-Roussillon | 6 (2) | 70 (5) |
| Limousin | 4 (1) | 24 (2) |
| Lorraine | 9 (2) | 48 (3) |
| Midi-Pyrénées | 20 (6) | 66 (4) |
| Nord-Pas de Calais | 28 (8) | 86 (6) |
| PACA | 22 (6) | 127 (8) |
| Pays de la Loire | 24 (7) | 78 (5) |
| Picardie | 11 (3) | 33 (2) |
| Poitou-Charentes | 7 (2) | 31 (2) |
| Rhône-Alpes | 38 (10) | 155 (10) |
| DOM - TOM | 12 (3) | 51 (3) |
| *France* | *361 (100)* |  |
| Belgium | 6 | NA |
| Switzerland | 9 | NA |
| Not available | 13 |  |
| Total | 389 |  |
